# Fats and Factors: Lipid Profiles Associate with Personality Factors and Suicidal History in Bipolar Subjects

**DOI:** 10.1371/journal.pone.0029297

**Published:** 2012-01-13

**Authors:** Simon J. Evans, Alan R. Prossin, Gloria J. Harrington, Masoud Kamali, Vicki L. Ellingrod, Charles F. Burant, Melvin G. McInnis

**Affiliations:** 1 Department of Psychiatry, University of Michigan, Ann Arbor, Michigan, United States of America; 2 School of Pharmacy, University of Michigan, Ann Arbor, Michigan, United States of America; 3 Department of Internal Medicine, University of Michigan, Ann Arbor, Michigan, United States of America; University of Adelaide, Australia

## Abstract

Polyunsaturated fatty acids (PUFA) have shown efficacy in the treatment of bipolar disorder, however their specific role in treating the illness is unclear. Serum PUFA and dietary intakes of PUFA associate with suicidal behavior in epidemiological studies. The objective of this study was to assess serum n-3 and n-6 PUFA levels in bipolar subjects and determine possible associations with suicidal risk, including suicidal history and relevant personality factors that have been associated with suicidality. We studied 27 bipolar subjects using the NEO-PI to assess the big five personality factors, structured interviews to verify diagnosis and assess suicidal history, and lipomics to quantify n-3 and n-6 PUFA in serum. We found positive associations between personality factors and ratios of n-3 PUFA, suggesting that conversion of short chain to long chain n-3s and the activity of enzymes in this pathway may associate with measures of personality. Thus, ratios of docosahexaenoic acid (DHA) to alpha linolenic acid (ALA) and the activity of fatty acid desaturase 2 (FADS2) involved in the conversion of ALA to DHA were positively associated with openness factor scores. Ratios of eicosapentaenoic acid (EPA) to ALA and ratios of EPA to DHA were positively associated with agreeableness factor scores. Finally, serum concentrations of the n-6, arachidonic acid (AA), were significantly lower in subjects with a history of suicide attempt compared to non-attempters. The data suggest that specific lipid profiles, which are controlled by an interaction between diet and genetics, correlate with suicidal history and personality factors related to suicidal risk. This study provides preliminary data for future studies to determine whether manipulation of PUFA profiles (through diet or supplementation) can affect personality measures and disease outcome in bipolar subjects and supports the need for further investigations into individualized *specific* modulations of lipid profiles to add adjunctive value to treatment paradigms.

## Introduction

Bipolar Illness strikes approximately 5.7 million adults in the United States, or about 1–2% of the adult population in any given year [1]. Importantly, the lifetime prevalence of suicide attempts in bipolar patients is over 30% [2], making uncontrolled bipolar disorder a large risk factor for suicidal behavior. In spite of the plethora of medications used to manage bipolar disease, including 60 years of clinical use of lithium, the majority of treated bipolar patients have “less than a satisfactory outcome” [3].

Several potential risk factors have been linked to suicidal behavior. Two of these include personality factors and PUFA serum levels. It is unknown whether PUFA serum levels are associated with personality factors and if these may interact to affect suicidal behavior. However, several studies suggest that low serum n-3 PUFA associate with aggressive and violent behavior. For example, n-3 PUFA have been found to be lower in suicide attempters [4] and violent suicides correlate with seasonal variation in n-3 intake [5]. Also, serum n-3 PUFA levels predict serotonin and dopamine metabolites in cerebrospinal fluid that differ between violent and non-violent subjects [6].

Supplementation with the long-chain n-3 (n-3) fatty acids, docosahexaenoic acid (DHA) and eicosapentaenoic acid (EPA), either as stand alone or adjunctive therapies have shown efficacy in the treatment of bipolar disorder. However, results across studies are inconsistent and the role of n-3s in the treatment of bipolar disorder is unclear. A 2008 review by Turnbull et al. surveyed clinical trials that used n-3s to treat bipolar disorder [7]. From over 100 publications, seven high quality and well-controlled trials were identified, of which four found positive mood improvements, two found no mood improvements and one did not report on mood improvements following n-3 supplementation. Therefore, while data is promising there remains ambiguity in the field. One factor not considered in the trials to date is the concentration of n-6 (n-6) fatty acids, which play complex physiological roles in both health benefit and pro-inflammatory state and are known to compete with n-3 fatty acids in many cellular processes, and therefore should be considered.

Evidence for the involvement of n-3 and n-6 fatty acids in bipolar disorder comes from several related lines of investigation. First, epidemiological studies have pointed to an association between n-3 and n-6 dietary intake and lifetime prevalence of bipolar disorder. Populations that consume greater long-chain n-3s and less long chain n-6s have a lower incidence of bipolar disease [8]. Second, animal studies have shown that diets deficient in n-3s alter monoamine systems in limbic structures known to control mood (reviewed by Chalon [9]), which provides a possible mechanistic link to psychiatric disorders. Third, mood stabilizers commonly used to treat bipolar disorder specifically inhibit membrane turnover and downstream signaling of the n-6 fatty acid, arachidonic acid (AA), but not the n-3, DHA [10]. Both lithium and carbamazepine decrease expression of phospholipase A2, responsible for cleaving AA from the membrane. Additionally, lithium, carbamazepine, valproate and lamotrigine inhibit COX2 expression, responsible for processing AA into the prostaglandin E2 series signaling molecules. These data suggest that mood stabilizers tip the scales in the direction of n-3 activity, by inhibiting AA function and reducing competition with DHA and EPA. Furthermore anti-depressants that increase AA activity in rodents (e.g. imipramine), have a higher probability of inducing mania in human bipolar subjects [11]. These data are consistent with the hypothesis that low n-3 or high n-6 tissue concentration may destabilize mood and suggest that the tissue balance between competing n-3 and n-6 fatty acid species may play a role in the pathoetiology of bipolar disorder.

**Figure 1:**
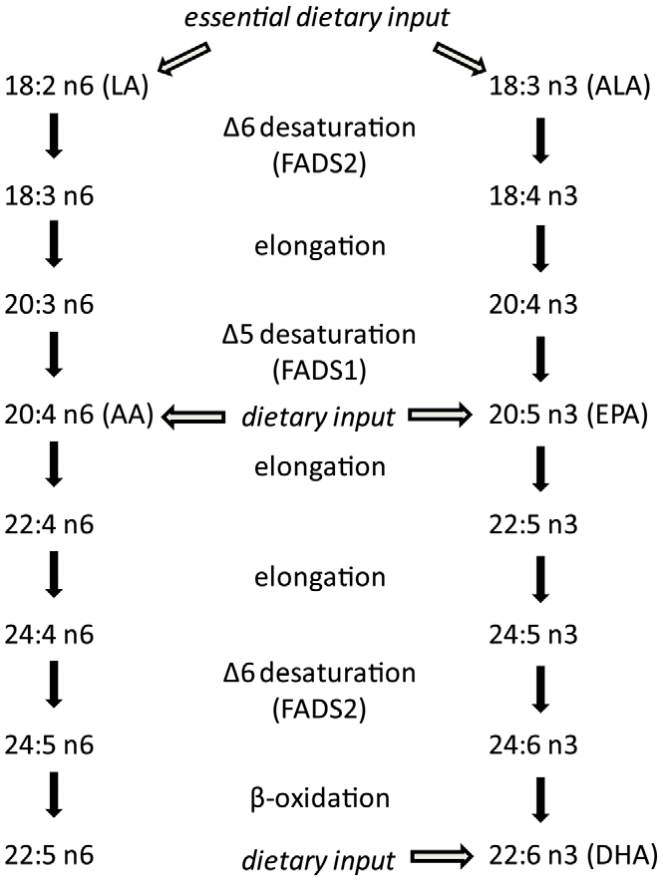
Schematic of biosynthetic pathway for common n-3 and n-6 fatty acids. Major dietary inputs are shown with arrows and enzymes catalyzing each conversion are given.

The n-3 and n-6 PUFA are essential in the diet, since they cannot be synthesized *de novo* by mammals. Technically, we only need to obtain the n-3, alpha linolenic acid (ALA), and the n-6, linoleic acid (LA), from dietary sources to synthesize longer chain n-3 and n-6 lipids from those (Figure 1). However, studies have shown that humans are inefficient at converting short to long chain fatty acids and thus the tissue composition of all n-3 and n-6 lipids partially reflects dietary consumption [12]. Single nucleotide polymorphisms (SNPs) in fatty acid desaturase (FADS) genes, partially responsible for metabolic inter-conversion, influence n-3 and n-6 serum levels as well [13], defining a diet x gene interaction in the control of fatty acid serum profiles and, potentially, factors related to psychiatric illness.

Following absorption, n-3 and n-6 fatty acids compete with each other for esterification and incorporation into cellular membranes, where they can affect the local microenvironment. In neurons, higher concentrations of DHA increase membrane fluidity and receptor kinetics [14]. The n-3, EPA, and the n-6, AA, also compete with each other for processing to various eicosanoids, which often have opposing physiological functions relative to immune inhibition or activation, respectively. Furthermore, the activity of the FADS1, FADS2 and elongase enzymes (depicted in Figure 1) can also control the available pools of short chain or long chain fatty acids, providing a level of competition within each of the n-3 or n-6 classes as well.

Therefore, to fully understand the efficacy of n-3 and n-6 fatty acids in bipolar depressive illness we will need an understanding of the subjects' complete lipid profiles and the ratios between competing lipids and their possible influence on various burdens of disease. The current report explores the relationship between PUFA profiles and personality factors, which themselves have associated with bipolar disorder and global functioning in bipolar subjects ([15], [16]). We analyzed plasma profiles of n-3 and n-6 fatty acid species in 27 well-characterized bipolar subjects and determined association with personality factors and suicidal history.

Given prior literature discussed above that n-3 intake inversely associates with violent behavior and suicidality, we hypothesize that serum levels of the long chain n-3s, DHA and EPA, may positively associate with personality factors that may be protective against suicide behavior and/or negatively associate with personality factors that, themselves, associate with increased risk of suicide behavior. We also hypothesize that the n-6s, linoleic acid and arachidonic acid, may modify the relationship between n-3s and psychiatric measures and that physiologically relevant ratios on n-3s and n-6 may be important to consider in the analyses.

## Results

In assessing the relationship between the five personality factor scores and n-3, n-6 fatty acids, we found that the relative plasma concentrations of several lipid species or physiologically relevant ratios between lipid species significantly correlated with various personality factors. Because of the pilot nature of this study, multiple testing corrections were not applied, however, all test are reported in Table 1. We found twice the number of significant correlations, below the 5% type I error threshold, than would be predicted by chance. However, independence of the tests cannot be assumed and we found a clustering of significant findings of associations between EPA (relative to it's precursor and product) and personality factor scores.

**Table 1:** Statistical Tests.

Factor	Statistic	LA	ALA	AA	EPA	DHA	AA:EPA	AA:DHA	LA:AA	ALA:EPA	ALA:DHA	EPA:DHA	FADS1	FADS2
N	Pearson (cor.)	0.40 (0.23)	0.06 (−0.01)	0.14 (0.25)	0.11 (0.18)	0.10 (0.05)	−0.17 (−0.22)	−0.09 (−0.02)	0.14 (−0.04)	−0.07 (−0.23)	−0.06 (−0.04)	0.09 (0.31)	0.07 (0.23)	0.18 (0.12)
	p-value (cor.)	**0.04 (0.28)**	0.76 (0.96)	0.48 (0.24)	0.58 (0.39)	0.61 (0.80)	0.38 (0.30)	0.66 (0.92)	0.49 (0.85)	0.74 (0.28)	0.78 (0.85)	0.65 (0.14)	0.75 (0.29)	0.38 (0.56)
	N (cor. df)	27 (22)	27 (22)	27 (22)	27 (22)	27 (22)	27 (22)	27 (22)	27 (22)	27 (22)	27 (22)	27 (22)	27 (22)	27 (22)
E	Pearson (cor.)	−0.07 (0.03)	0.06 (0.04)	0.17 (0.21)	0.28 (0.18)	0.08 (0.02)	−0.27 (−0.21)	−0.17 (−0.14)	−0.17 (−0.15)	−0.39 (−0.32)	−0.18 (−0.14)	0.38 (0.28)	−0.11 (−0.13)	0.03 (0.12)
	p-value (cor.)	0.73 (0.88)	0.78 (0.87)	0.40 (0.33)	0.16 (0.40)	0.70 (0.94)	0.17 (0.32)	0.39 (0.52)	0.40 (0.47)	**0.04 (0.13)**	0.38 (0.52)	**0.05 (0.18)**	0.58 (0.55)	0.88 (0.58)
	N (cor. df)	27 (22)	27 (22)	27 (22)	27 (22)	27 (22)	27 (22)	27 (22)	27 (22)	27 (22)	27 (22)	27 (22)	27 (22)	27 (22)
O	Pearson (cor.)	0.09 (−0.02)	0.07 (0.05)	0.20 (0.26)	0.10 (0.15)	0.35 (0.38)	−0.08 (−0.07)	−0.26 (−0.24)	−0.11 (−0.22)	−0.24 (−0.30)	−0.50 (−0.51)	−0.17 (−0.16)	−0.09 (−0.03)	0.41 (0.41)
	p-value (cor.)	0.66 (0.92)	0.74 (0.82)	0.31 (0.22)	0.62 (0.49)	0.07 (0.07)	0.69 (0.73)	0.19 (0.27)	0.57 (0.30)	0.24 (0.15)	0.01 (0.01)*	0.39 (0.45)	0.66 (0.88)	0.04 (0.04)*
	N (cor. df)	27 (22)	27 (22)	27 (22)	27 (22)	27 (22)	27 (22)	27 (22)	27 (22)	27 (22)	27 (22)	27 (22)	27 (22)	27 (22)
A	Pearson (cor.)	−0.08 (−0.07)	−0.09 (−0.14)	−0.00 (0.04)	0.11 (−0.03)	0.00 (−0.08)	−0.21 (−0.15)	0.05 (0.12)	−0.12 (−0.14)	−0.44 (−0.41)	−0.08 (−0.04)	0.43 (0.39)	0.01 (0.04)	−0.09 (−0.04)
	p-value (cor.)	0.69 (0.76)	0.65 (0.51)	0.99 (0.87)	0.60 (0.91)	0.99 (0.70)	0.29 (0.48)	0.80 (0.57)	0.54 (0.51)	0.02 (0.05)*	0.67 (0.87)	0.02 (0.06)*	0.96 (0.86)	0.64 (0.85)
	N (cor. df)	27 (22)	27 (22)	27 (22)	27 (22)	27 (22)	27 (22)	27 (22)	27 (22)	27 (22)	27 (22)	27 (22)	27 (22)	27 (22)
C	Pearson (cor.)	−0.07 (−0.01)	−0.02 (−0.13)	−0.03 (−0.03)	0.30 (0.11)	0.39 (0.27)	0.11 (0.19)	−0.06 (0.01)	−0.07 (−0.04)	−0.18 (−0.12)	−0.35 (−0.33)	−0.10 (−0.34)	−0.13 (−0.15)	0.22 (0.29)
	p-value (cor.)	0.71 (0.95)	0.91 (0.53)	0.90 (0.89)	0.12 (0.61)	0.04 (0.20)	0.58 (0.38)	0.77 (0.96)	0.74 (0.86)	0.38 (0.59)	0.07 (0.12)	0.62 (0.11)	0.52 (0.47)	0.28 (0.16)
	N (cor. df)	27 (22)	27 (22)	27 (22)	27 (22)	27 (22)	27 (22)	27 (22)	27 (22)	27 (22)	27 (22)	27 (22)	27 (22)	27 (22)

Factors: N, Neuroticism; E, Extraversion; O, Openness; A, Agreeableness; C, Conscientiousness

Correlations and p-values are shown in each box with corrections for age, BMI and antipsychotic dose shown in the parentheses. Significant correlations are bolded with those remaining significant after correction marked with an asterisk.

**Figure 2:**
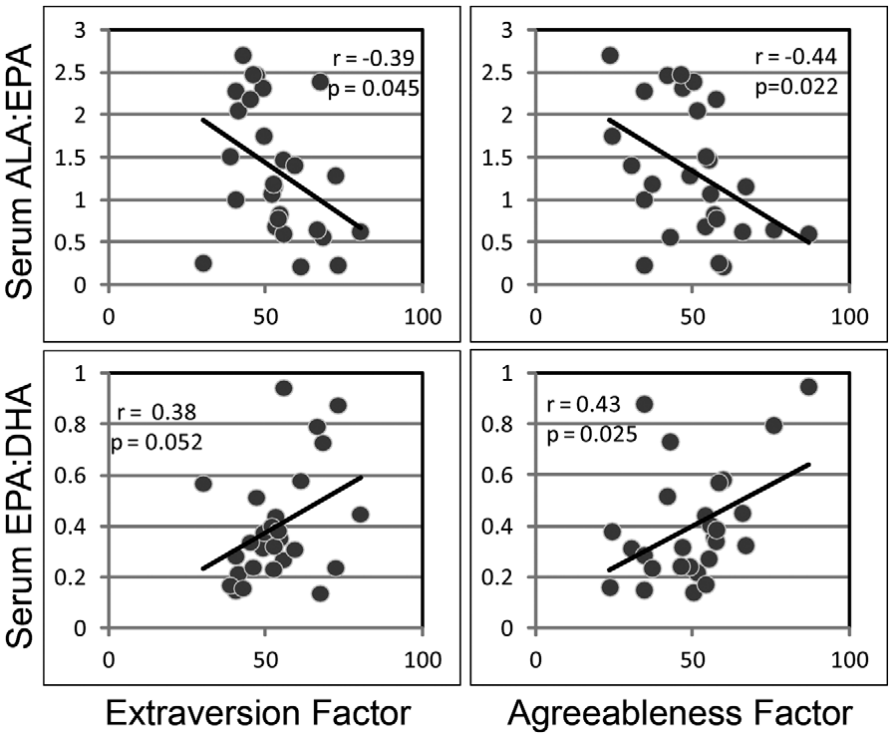
Scatter plots of ALA:EPA ratio, EPA:DHA ratio and both agreeableness and extraversion factor scores. Graphs show significant correlations between ALA:EPA and agreeableness (p = 0.023, r = −0.436) and extraversion (p = 0.045, r = −0.389) and significant positive correlations between EPA:DHA and agreeableness (p = 0.024, r = 0.433) and extraversion (p = 0.048, r = 0.384).

Figure 2 shows Pearson correlations revealing that the ALA:EPA ratio negatively correlated and the EPA:DHA ratio positively correlated with extraversion (p = 0.045, r = −0.389 for ALA:EPA; p = 0.048, r = 0.384 for EPA:DHA) and agreeableness (p = 0.023, r = −0.436 for ALA:EPA; p = 0.024, r = 0.433 for EPA:DHA). These data suggest that a higher pool of EPA, relative to its precursor, ALA, and its downstream product, DHA, associate with extraversion and agreeableness. The correlations with agreeableness remained significant following correction for age, BMI and antipsychotic medication dose.

**Figure 3:**
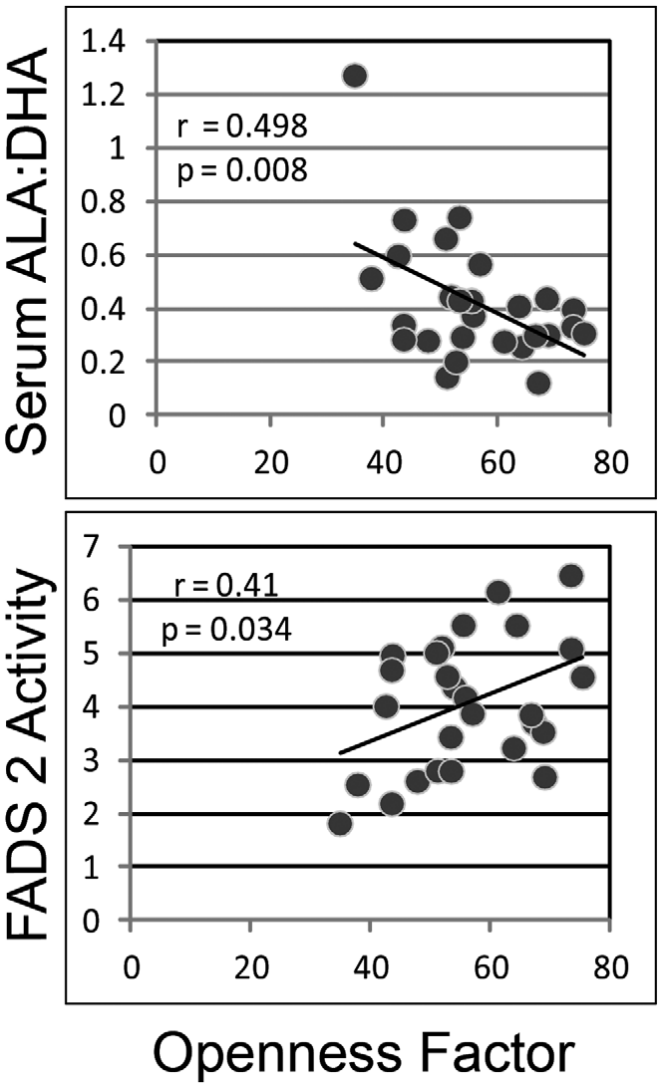
Scatter plot of openness factor score and (a) serum ALA:DHA ratio or (b) FADS2 activity. Graphs show a significant negative correlation between openness factor score and serum ALA:DHA ratio (p = 0.008, r = −0.498) and a significant positive correlation between openness factor score and FADS2 activity (p = 0.036, r = −0.406).

Figure 3 shows that the ALA:DHA ratio negatively correlated with openness factor score (p = 0.008, r = −0.498) and FADS2 activity positively correlated with openness factor score (p = 0.036, r = −0.406). These data suggest that more efficient conversion of the 18 carbon ALA to the 22 carbon DHA (partially dependent on FADS2 activity) may associate with increased openness to experience. These data remained significant following correction for age, BMI and antipsychotic medication dose (Table 1).

**Figure 4:**
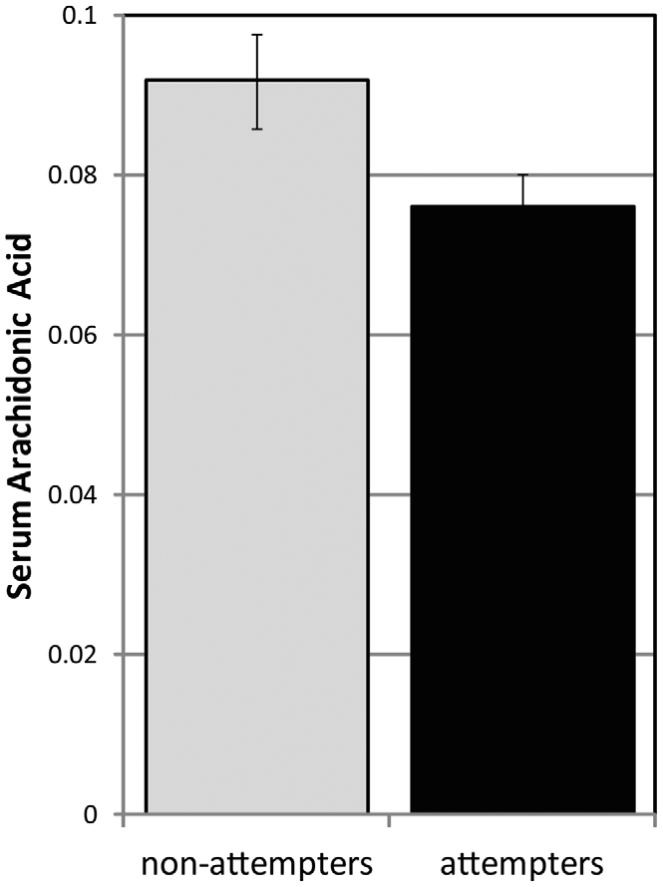
Bar graph of differences in serum AA concentration between subjects with and without a history of suicide attempt. Serum AA is plotted for both suicide attempters and non-attempters; error bars show standard deviation (p = 0.026).

In an analysis that compared lipid species in bipolar patients with and without a history of suicide, we found a significantly lower serum AA concentration (p = 0.026) and a trend for lower EPA serum concentrations (p = 0.070) in suicide attempters, as shown in figure 4a. After correcting for age, BMI and antipsychotic medication use, the difference in AA serum concentration between the suicide attempters and non-attempters remained significant (p = 0.037).

## Discussion

Identifying metabolic or dietary factors that influence factors associated with psychiatric illness may provide a path to improving therapeutic tools. In previous studies, an effect of n-3 fatty acid supplementation has shown inconsistent association with improvements in bipolar symptomology ([7]) and we suggest that this may be due to variable concentrations of other lipid species, either n-6 fatty acids that tend to compete with n-3 fatty acids in a variety of signaling, inflammatory and other pathways; or genetically variant enzyme activities that favor pooling one fatty acid over another. In this report, we found that n-3 and n-6 lipid profiles correlate with aspects of the five factor personality model that have themselves been associated with suicidal behavior ([16], [17], [18]). This does not imply a relationship between PUFA profiles and suicide. However, given prior literature on such a potential relationship ([19]), the current study contributes to a warranting of further investigation into the relationship between the various components of suicide risk and PUFA profiles.

Without a control population in the current study, it is impossible to state whether or not the association between PUFA profiles and personality factors is specific to a psychiatric population. However, this is not our purpose. If PUFA profiles influence personality factors, like agreeableness and extraversion, this may be one additional risk factor for suicidal behavior in psychiatric patients, like bipolars, who are already at increase risk for suicide. An association between PUFA profiles and personality factors may also exist in a healthy population, but without significant consequence. Larger studies with a control population are necessary to answer these questions.

We also found associations between lipid profiles and suicidal history in bipolar subjects. These data further support a link between essential fatty acid metabolism and mood disorders. While the current pilot study is an observational, cross-sectional study, it raises important questions regarding potential causative roles for lipid profiles in regulating personality phenotypes that may impact the treatment of bipolar disorder. We are cognizant of the fact that personality factors, promoted as trait markers in bipolar disorder are not entirely stable [20] and longer-term longitudinal studies are necessary to examine the relationship between personality traits and fatty acid profiles.

In the current study, we found that DHA:ALA ratios and FADS2 activity (which promotes DHA synthesis) significantly correlated with openness personality factors, correlations that remained significant following correction for age, BMI and antipsychotic medication dose. These data suggest that the conversion from short chain ALA to long chain DHA, which is moderated by both dietary and genetic factors that control FADS1 and FADS2 activity, associate with openness, which itself is positively associated with suicidal risk in bipolar subjects [21]. Therefore, this provides one point where dietary input and n-3 metabolism may interact to contribute to the regulation of personality factors that influence suicidal behavior. Future prospective studies are required to clarify whether there is a causal role for dietary PUFA that can be manipulated to reduce suicide risk.

In support of this EPA:ALA and EPA:DHA ratios correlated with agreeableness and this association also remained significant following correction for age, BMI and antipsychotic medication dose. Thus, a higher pool of EPA relative to its precursor ALA or its product DHA, which would be promoted by *lower* FADS2 activity, associated with higher agreeableness factor scores. Previously, higher agreeableness scores have been shown to be protective against suicidal behavior [18].

Finally, we found that the long chain n6, AA, was lower in subjects with a history of suicide attempt, suggesting that increased serum concentrations of this PUFA may be protective. This is consistent with prior literature associating suicidal behavior with decreased serum lipids, including cholesterol, although this is controversial [22]. Higher AA pools would also be promoted by lower FADS2 activity, since this enzyme promotes the conversion of AA to longer chain lipids. These data may seem paradoxical since one would expect the activity of the n-3 fatty acids, EPA and DHA to be lower due to competition with increased AA. However, in an analysis that looked at AA to DHA, AA to EPA and total n-6 to total n-3 ratios, we found that all of these ratios were higher in subjects with a history of suicide, suggesting a complex relationship between absolute and relative AA concentration as a risk factor for psychiatric illness. These data are not presented because they did not reach statistical significance, potentially due to the low power of the current study.

Taken together, these data suggest that increased activity of FADS2, which is genetically moderated with known SNP variants that control serum lipid profiles [13], may increase the risk of suicidal behavior in bipolar subjects. Referring to figure 1, increased activity of FADS2 would reduce the pool of both AA and EPA by promoting conversion to longer chain fatty acids shown in both the n-3 and n-6 pathways. We found the increased FADS2 activity and several consequences of increased activity, including increased DHA:ALA, increased DHA:EPA, decreased EPA:ALA and reduced AA serum concentrations, all associated with markers of increased suicidal risk, as discussed above. Interestingly, increased FADS2 gene expression has been previously reported in the prefrontal cortex of bipolar subjects [23], a brain region that influences mood circuitry.

Limitations of this study include a small sample size, a clinically diverse group (regarding current status and medication history) and the lack of a healthy control group to make comparisons, which all limit the statistical power of the study and ability to extrapolate to a larger population. Furthermore, the lack of multiple testing correction requires the data be examined cautiously and the need for confirmation in larger sample sets with the added power to analyze SNP variants directly. This will be the focus of our future investigations. However, our results focused on EPA are consistent with prior literature implicating this PUFA in mood regulation and raises interesting questions as to whether specific SNPs in the FADS genes and other lipid metabolism control points may be useful in guiding PUFA dietary or supplementation strategies that could augment the treatment of bipolar patients through dietary approaches.

## Materials and Methods

Bipolar disorder subjects, receiving an atypical antipsychotic (olanzapine, quetiapine, risperidone or clozapine) were recruited for this cross-sectional study, as reported previously [24]. After informed consent was obtained, subjects underwent metabolic screening and fasted blood samples were drawn and used for lipomic analysis. All recruitment, enrolling and data analysis were done with the approval of the Institutional Review Board for human subject use at the University of Michigan and written consent for the studies was obtained from all subjects. Medical and demographic data for the subjects are summarized in Table 2, separated into suicide attempter and non attempter groups.

**Table 2:** Subject Data.

Age	Gender	Current AAP	Dose (mg/day)	CPZ eqs.	BMI	Suicide Hx
53	F	Quetiapine	300	170.9	25	No
51	F	Quetiapine	12.5	7.1	25	No
57	F	Quetiapine	600	341.9	21	No
64	M	NA	NA	0.0	32	No
26	F	Aripiprazole	5	62.7	22	No
56	F	Aripiprazole	20	250.9	45	No
51	F	Quetiapine	400	227.9	33	No
45	M	Quetiapine	600	341.9	38	No
58	F	Quetiapine	100	57.0	33	No
61	F	Risperidone	4	332.1	29	No
22	F	Aripiprazole	7.5	94.1	22	Yes
47	F	Clozapine	200	144.1	26	Yes
33	F	Paliperidone	3		38	Yes
54	M	Aripiprazole	15	188.2	33	Yes
50	M	Aripiprazole	25	313.6	24	Yes
25	F	Risperidone	2	166.0	21	Yes
25	F	NA	NA	0.0	39	Yes
42	F	NA	NA	0.0	20	Yes
60	M	Quetiapine	125	71.2	30	Yes
42	M	Risperidone	2	166.0	31	Yes
38	M	Olanzapine	7.5	140.7	30	Yes
37	F	Quetiapine	25	14.2	35	Yes
56	F	Risperidone	1	83.0	33	Yes
42	F	Olanzapine	15	281.5	35	Yes
56	F	NA	NA	0.0	29	Yes
54	F	NA	NA	0.0	43	Yes
35	F	Ziprasidone	160	255.5	23	Yes

Atypical antipsychotic (AAP) medications are given with actual dose and chlorpromazine equivalents (CPZ eqs.). Body mass index (BMI) and history of suicide attempt (Suicidal Hx), either Yes or No are also reported.

Total fasted plasma lipid profiles were obtained from 27 bipolar subjects who were dual enrolled in a second study, for which detailed psychiatric data were available. Total lipids were extracted from the plasma according to the method of Bligh and Dyer [25]. Heptadecanoic acid internal standard for lipid sub-classed was added to each sample prior to extraction. After hydrolysis, lipids were methylated and analyzed on an Omega Wax 250 capillary column (Supelco) using an Agilent 6890 gas chromatograph. Relative abundance of 22 different naturally occurring fatty acid and 3 trans-fatty acid species were done by comparison of retention times with known standards. FADS2 activity estimates were calculated using the ratio of 18∶3 (n-6) to 18∶2 (n6).

The revised NEO personality inventory (NEO-PI-R) was used to measure the five personality factor scores (neuroticism, extraversion, openness, agreeableness and conscientiousness). We chose to normalize the NEO-PI-R data to the S-Form and all factors were calculated as per the NEO-PI-R manual [26]. (NEO PI-R professional manual. Odessa, FL: Psychological Assessment Resources, Inc.). Suicidal history was extracted from a clinician directed interview using the Diagnostic Interview for Genetic Studies (DIGS, [27]). All subjects were also completed a Young Mania Rating Scale and a Hamilton Depression Scale on the same day as the NEO-PI. Twenty one subjects showed no signs of mania (score<8), 4 showed a slight increase in mania (score 9–11), one showed moderate mania (score = 16) and one showed elevated mania (score = 33). Fifteen showed no depression (score<8), 8 showed minor depression (score 9–15), and 4 showed moderate depression (score 16–20).

Data were analyzed with SPSS 19 software (IBM) using a bivariate correlation analysis for the lipid concentration - personality factor score correlations; and linear multivariate analysis for group comparisons between suicide attempter and non-attempter groups, using age, BMI and antipsychotic medication dose (normalized to chlorpromazine equivalents by the method of Andreasen, et al. [28]) as covariates in the model.
